# Could Natural Products Help in the Control of Obesity? Current Insights and Future Perspectives

**DOI:** 10.3390/molecules28186604

**Published:** 2023-09-13

**Authors:** Jiwon Park, Fahrul Nurkolis, Hyunji Won, Jiye Yang, Dayeon Oh, Hyunkyung Jo, Jinwon Choi, Sanghyun Chung, Rudy Kurniawan, Bonglee Kim

**Affiliations:** 1 Department of Pathology, College of Korean Medicine, Kyung Hee University, Hoegidong Dongdaemun-gu, Seoul 02447, Republic of Korea; 2 Department of Biological Sciences, State Islamic University of Sunan Kalijaga (UIN Sunan Kalijaga), Yogyakarta 55281, Indonesia; fahrul.nurkolis.mail@gmail.com; 3 Kyung Hee Myungbo Clinic of Korean Medicine, Hwaseong-si 18466, Republic of Korea; 4 Diabetes Connection Care, Eka Hospital Bumi Serpong Damai, Tangerang 15321, Indonesia; 5 Korean Medicine-Based Drug Repositioning Cancer Research Center, College of Korean Medicine, Kyung Hee University, Seoul 02447, Republic of Korea

**Keywords:** obesity, natural products, herbal medicine, lipid metabolism, antioxidant, anti-inflammation, bioactive molecules, metabolic syndrome

## Abstract

Obesity is a global issue faced by many individuals worldwide. However, no drug has a pronounced effect with few side effects. Green tea, a well-known natural product, shows preventive effects against obesity by decreasing lipogenesis and increasing fat oxidation and antioxidant capacity. In contrast, other natural products are known to contribute to obesity. Relevant articles published on the therapeutic effect of natural products on obesity were retrieved from PubMed, Web of Science, and Scopus. The search was conducted by entering keywords such as “obesity”, “natural product”, and “clinical trial”. The natural products were classified as single compounds, foods, teas, fruits, herbal medicines—single extract, herbal medicines—decoction, and herbal medicines—external preparation. Then, the mechanisms of these medicines were organized into lipid metabolism, anti-inflammation, antioxidation, appetite loss, and thermogenesis. This review aimed to assess the efficacy and mechanisms of effective natural products in managing obesity. Several clinical studies reported that natural products showed antiobesity effects, including *Coffea arabica* (coffee), *Camellia sinensis* (green tea), *Caulerpa racemosa* (green algae), *Allium sativum* (garlic), combined *Ephedra intermedia* Schrenk, *Thea sinensis* L., and *Atractylodes lancea* DC extract (known as Gambisan), *Ephedra sinica* Stapf, Angelica Gigantis Radix, Atractylodis Rhizoma Alba, *Coicis semen*, *Cinnamomi cortex*, *Paeoniae radix* alba, and *Glycyrrhiza uralensis* (known as Euiiyin-tang formula). Further studies are expected to refine the pharmacological effects of natural products for clinical use.

## 1. Introduction

Obesity is an excessive accumulation of fat, which poses a potential health risk. Specifically, a body mass index (BMI) of >30 is considered obese [1,2]. Currently, >1 billion individuals are obese globally [3]. This number is still increasing [3], meaning that an increasing number of individuals are becoming susceptible to many serious diseases, such as hypertension, dyslipidemia, type 2 diabetes, coronary heart disease, stroke, osteoarthritis, and cancer, due to this chronic and relapsing disease [4].

One typical treatment for obesity is weight loss drugs approved by the US Food and Drug Administration, including orlistat, phentermine-topiramate, and naltrexone-bupropion. Chemical medications can help lose weight and maintain weight loss but can also cause changes in behavior [5]. However, weight-loss drugs have been withdrawn from the market because of side effects [6]. Among those still in use, orlistat, naltrexone-bupropion, phentermine-topiramate, liraglutide, and semaglutide have been used for long-term treatment. In contrast, others are only used for short-term treatment due to unguaranteed safety over longer periods [5]. Even these drugs may show adverse effects in some individuals and can be inaccessible because of high prices [6].

Therefore, developing new drugs, including botanical drugs, phytomedicines, traditional medicines, and herbal medicines, has gained importance. They have been suggested as substitutes for chemical drugs to reduce side effects while maintaining effectiveness. For example, *Ephedrae herba* showed preventive effects against hyperlipidemia in mice, possibly by regulating DNA repair and modulating the expression of genes and proteins related to energy metabolism [7].

Therefore, this study aimed to identify natural products that are effective against obesity and examine their effects. After listing the substances tested in clinical trials, we divided them into seven groups: single compound, food, tea, fruit, herbal medicine—single extract, herbal medicine—decoction, and herbal medicine—external preparation. The related studies’ results were examined to estimate each group’s effectiveness.

## 2. Methods

Relevant articles published between 2016 and 2022 on the therapeutic effect of natural products on obesity were retrieved from PubMed, Web of Science, and Scopus. The search was conducted using keywords such as “obesity”, “natural product”, and “clinical trial”. More than one hundred studies were retrieved. We excluded reviews, duplicate articles, studies not written in English, studies with an English abstract but no English full text, studies on patients with obesity but not treating obesity, and studies reporting no significant effect on obesity. Overall, 66 studies demonstrating the efficacy of using natural products to treat obesity were selected for this review.

## 3. Obesity Treating Natural Products

Many published studies show that diverse natural products are effective in treating obesity. In this review, natural products were classified into five categories based on the form of the experimental drug: single compound, food, tea, fruit, and herbal medicine. Then, herbal medicine was reclassified into three subcategories based on the type used in the experiments: single extract, decoction, and external preparation.

### 3.1. Single Compound

One study with a single compound showed antiobesity effects (Table 1). Diethyl azelate (DEA) is naturally produced in animals and plants and can be used to improve related metabolic syndromes [8]. Steeper et al. reported that daily oral DEA decreased total cholesterol (TC) and low-density lipoprotein (LDL) levels in human males who were overweight, alleviating obesity. This study on DEA included 17 participants and lasted for 21 days. More reliable results would have been drawn if this study had enrolled more subjects. This study’s design also decreased its reliability; it used a 21-day prospective design in a before–after clinical trial and did not use blinding or a placebo control during treatment.

It was impossible to determine the trend of studies regarding the antiobesity effects of single compounds because there was only one study in this category.

### 3.2. Foods

Twenty-six human studies examined using foods to treat obesity (Table 2). 

*C. carvi* L. (caraway) aqueous extract (CAE) decreased WC, waist-to-hip ratio (WHR), thigh circumference (THC), and mid-upper arm circumference [12]. Rondanelli et al. demonstrated that *C. scolymus* (artichoke) decreased visceral adipose tissue (VAT), fat mass (FM), and WC [14]. These results demonstrated that artichokes could potentially treat individuals with overweight and impaired fasting glucose. *V. vinifera* L. (grape) seed extract (GSE) decreased several anthropometric measurements, including BW, BMI, WC, hip circumference (HC), and WHR, demonstrating its potential to treat obesity [16]. The treatment group received GSE (300 mg/day) for 12 weeks, also lowering neuropeptide Y (NPY) levels compared to the placebo group. An *L. plantarum* fermented barley–wheat flour compound noodle (FBWN) decreased WC, fat rate, FM, and visceral fat (VF) and increased muscle mass and basal metabolic rate [17]. Boix-Castejón et al. reported that combining *L. citriodora* (lemon beebrush) and *H. sabdariffa* (roselle; LC-HS) decreased appetite and attraction to fatty, sweet, and salty foods, decreasing obesity [18].

Matured *H. lupulus* L. (hop) bitter acids attenuated diet-induced body fat (BF) accumulation in rodents by enhancing thermogenesis in brown adipose tissue (BAT) through the activity of sympathetic nerves innervating BAT [19]. Morimoto-Kobayashi et al. reported that matured hop extract reduced total fat area primarily by reducing VF area (VFA). Oniki et al. reported that *G. gnemon* Linn. (melinjo) seed extract (MSE) activated genes regulating APN multimerization. It has been demonstrated that APN may enhance insulin sensitivity and protect against obesity, type 2 diabetes, and atherosclerosis [20]. Rao et al. reported that *N. sativa* (black seed or jintan hitam) and *Trigonella foenum-graecum* (fenugreek) supplemented chapatis (NFCs) also decreased BM, BMI, WC, HC, and the central obesity index [21]. Kim et al. reported that *P. grandiflorus* (balloon flower) ethanol extract (PGE) reduced BFM and BF percentage (BFP) [23]. PGE571 (PGE at 571 mg) decreased leptin levels, BFM, and BFP and increased muscle mass. PGE2855 (PGE at 2855 mg) decreased the leptin:APN ratio, BFM, BFP, and total abdominal and subcutaneous fat areas.

Nishimura et al. reported that quarantine-rich onion did not decrease the VFA [24]. Nevertheless, participants with low HDL-C levels in the quercetin-rich onion group showed significantly lower VFAs. Amini et al. reported that *S. officinalis* (common sage) decreased BW, BMI, and WC [25]. Common sage extract at 330 mg/day for eight weeks positively affected lipid metabolism. Maia-Landim et al. reported that standardized *G. cambogia* (Malabar tamarind) extracts (52.4% hydroxycitric acid (HCA)) and *A. konjac* (konjac; 94.9% glucomannan) decreased plasma glucose, cholesterol, and TG levels; FM; VF; and BW and increased the basal metabolic rate [26]. However, polymorphisms in perilipin 4 (*PLIN4*; −11482G > A), FM and obesity-associated (*FTO*; rs9939609 (A/T)), and β-adrenergic receptor 3 (*ADRB3*; Trp64Arg) attenuated its lipolysis effect. Farhat et al. reported that *S. rebaudiana* (stevia) intake did not result in energy compensation during lunch or throughout the day and reduced postprandial glucose levels compared to sugar [27]. Stevia was found to lower appetite and stop the increase in food intake. Leverrier et al. reported that 500 mg/day of *H. annuus* (sunflower) seed extract for 12 weeks decreased cholesterol, long-lasting LDL, BW, BMI, and WC [28]. The intervention was especially effective in females with obesity aged >30 years.

Six weeks of *C. lanatus* (watermelon) supplementation increased fasting plasma L-arginine, cis-lycopene, and trans-lycopene levels and decreased vascular cell adhesion molecule 1 (VCAM1) levels [29]. This study only suggested indirect effects on obesity, so further research is needed to obtain effective results for lipid metabolism. A new comprehensive study by Permatasari et al. showed that *C. racemosa* (green seaweed or green algae) could be a new candidate for antiobesity functional food [30]. This study integrated in silico and in vitro experiments with a four-week, randomized, double-blind, placebo-controlled clinical trial. A randomized, double-blind, parallel-group, placebo-controlled pilot study by Majeed et al. demonstrated the antiobesity potential of *C. rotundus* extract (CRE) [31]. Interestingly, CRE showed antiadipogenic activity, was safe for human consumption, and effectively managed weight and hypercholesterolemia in individuals with overweight.

The main active ingredient in Malabar tamarind extract is HCA, which is known to attenuate weight gain and fat synthesis in animals and humans [32]. However, the mechanism underlying the action of HCA is not fully understood. A three-month clinical study on 100 individuals with obesity and a subsequent computational study investigated the effect of HCA treatment on anthropometric measurements and plasma lipid profiles in human subjects [32]. They showed that HCA could reduce weight gain and fat accumulation in subjects with obesity. Han et al. conducted a randomized, double-blind, placebo-controlled study assessing the effect of standardized *H. serrata* (Thunb.) Ser. leaf extract (WHS) on BW and BF reduction in human subjects with overweight or obesity [33]. Daily WHS supplementation reduced BW, BMI, and BFM. Interestingly, this was accompanied by reduced HC, VFA, abdominal fat area, and the visceral–subcutaneous ratio. More interestingly, no significant side effects were observed during or after 12 weeks of this intervention. 

All the above studies support the claim that certain foods help prevent obesity. Foods used in these studies were usually also treated as herbal medicines, and many processed foods into extracts to test their effects on obesity. Certain foods reduce obesity usually by controlling metabolic hormones or reducing appetite. Most studies stated that there were no side effects. However, some studies used noodle or snack forms to test the food’s antiobesity effect [13,17]. Moreover, some studies did not clearly indicate a mechanism for reducing obesity. Therefore, further studies are needed.

### 3.3. Teas

Twelve human studies treated obesity using tea (Table 3). Yonekura et al. conducted a cross-sectional study on *C. arabica* (coffee) and *C. sinensis* (green tea). These substances were administered to 232 Japanese women aged 40–65 years with menopausal symptoms who completed the brief-type self-administered diet history questionnaire [35]. Patients were divided into four groups depending on their coffee (CF) and green tea (GT) consumption. Using a multivariate model, they showed an inverse relationship between daily CF/GT intake and BW, BMI, and cardio-ankle vascular index. Ghasemi et al. conducted a clinical trial using combined high-intensity interval training and green tea supplementation in 30 women with overweight [36]. They determined that daily green tea consumption increased the levels of sirtuin 1 (SIRT1), peroxisome proliferator-activated receptor gamma coactivator 1-alpha (PGC-1α), and catalase (CAT) and significantly decreased BFP, BMI, and BW. Therefore, the catechins in green tea inhibit lipogenesis, increase fat oxidation, and improve antioxidant capacity. Kobayashi et al. conducted a randomized, double-blind, placebo-controlled trial examining the effectiveness of green tea beverages enriched with catechins and a galloyl moiety on obesity in 124 subjects with obesity [37]. Green tea catechins with a galloyl moiety reduced BW, BMI, and BFP by decreasing abdominal fat area via inhibiting or attenuating intestinal fat absorption.

All these studies support the view that tea is effective in weight loss. Most studies supported green tea’s ability to help individuals lose weight; only one study found the beverage ineffective. Therefore, further research on the obesity-reducing effect of green tea is needed. In addition to green tea, coffee, kosen-cha, oolong tea, and puer tea were reported to alleviate obesity.

### 3.4. Fruits

Six studies demonstrated the effectiveness of fruit-derived natural products in ameliorating obesity (Table 4). Duchnowicz et al. reported that *A. melanocarpa* decreased acetylcholinesterase (AChE) activity and oxidative stress, improving lipid metabolism related to cholinesterase activity [46]. *A. melanocarpa* at 3 × 100 mg/day for two months decreased cholesterol and lipid peroxidation, reducing AChE. Rondanelli et al. found that bergamot phytosome positively affected VAT after 30 days and remained effective for a further 60 days [47]. Bergamot phytosome tablets (500 mg) taken twice daily for 12 weeks modulated lipids, decreasing TC and LDL and increasing HDL. All these studies support the efficacy of fruit-derived natural products against obesity and lipid disorders, although there were some limitations. Treatments in several studies appeared effective but were not significant. In addition, a few studies were conducted on obesity-related bioavailability, such as metabolic disorders, inflammatory status, and antioxidant capacity, rather than on obesity itself.

### 3.5. Herbal Medicines

Herbal medicines have been used to treat various diseases in East Asia for millennia, of which several have antiobesity effects. Here, we divide herbal medicines into three categories: single extracts, decoctions, and external preparations.

#### 3.5.1. Herbal Medicines—Single Extracts

Eight studies examined the antiobesity effects of herbal medicine—single extracts (Table 5). *S. maxima* extract was reported to influence lipid profiles due to its correlation with reduced LDL [53], providing encouraging results in individuals with obesity when given at 2 g/day for three months. Yousefi et al. compared the effects of *S. platensis* powder to a control treatment, finding it decreased appetite, BW, BF, BMI, WC, and TG [54]. Improvements in individuals with obesity-associated metabolic disorders were noted after 12 weeks of treatment with one *S. platensis* tablet (2 g) daily. A 12-week study examined the beneficial effects of combining *Z. multiflora* (ZM) with oxymel on obesity in three groups: 0.75 g ZM in 10 mL oxymel, 1.5 g ZM in 10 mL oxymel, and 10 mL oxymel without ZM [55]. It showed reduced WC in all groups, while group A also showed reduced HC, and group B also showed a reduced waist-to-hip circumference ratio.

Altogether, these studies provide evidence supporting the antiobesity effects of herbal medicines—single extracts, although they showed similar limitations, including small sample sizes and short observation periods. In addition, only a few studies examined safety and tolerability. Therefore, further follow-up studies are needed to confirm their findings.

#### 3.5.2. Herbal Medicines—Decoctions

Eight studies suggested that herbal medicine decoctions have antiobesity effects in human subjects (Table 6 and Table 7). Cheon et al. reported that Euiiyin-tang could significantly reduce weight in patients with obesity after 12 weeks of treatment [60]. While both showed reduced weight, the decrease in WC and HC was greater in the Euiiyin-tang group than in the placebo group. Cho et al. reported that YY-312, an herbal extract powder from *I. cylindrica* Beauvois, *C. unshiu* Markovich, and *E. officinalis* Dode, reduced BF. Administrating 2400 mg/day of YY-312 for 12 weeks significantly reduced BFM, BFP, BW, and WC compared to the placebo [61]. Herranz-López et al. investigated the effects of subjects with overweight consuming a combination of polyphenolic LC-HS extracts enriched in polyphenols at a daily dosage of 500 mg for two months while maintaining an isocaloric diet [62]. The subjects showed meaningful reductions in BW, abdominal circumference, and BFP. Kudiganti et al. showed that taking 400 mg of Meratrim twice daily for 16 weeks significantly reduced BW, BMI, waist size, and hip size compared to the placebo without supplement-related AEs [63]. Dixit et al. reported that receiving 900 mg/day of LI85008F over two doses for 16 weeks significantly reduced BW and BMI compared to the placebo [64]. WC, HC, and WHR were also meaningfully reduced. Chung et al. concluded that patients treated with 900 mg/day of Qingxue Dan for eight weeks significantly reduced BMI and TG, with decreases in total BF, abdominal FM (AFM), and WC also noted [65]. Adamska-Patruno et al. found that combining *M. alba* (white mulberry), *P. vulgaris* (white bean), and *C. arabica* (green coffee) extracts decreased the adverse effects of high-glycemic index/load meal consumption [66]. Lower glucose and insulin levels were observed with both IP-A (a mixture of 400 mg green coffee, 600 mg white mulberry, and 1200 mg white bean extracts) and IP-B (a mixture of 400 mg green coffee, 600 mg white mulberry, and 1200 mg white bean extracts supplemented with 2000 mg inulin and 3000 mg glucomannan) treatments.

All these studies commonly suggest that herbal medicine decoctions can reduce obesity. Most studies used BW, BMI, and BFP as indicators of this reduction. However, some did not find significant results for some indicators. Therefore, further studies are needed to confirm their findings.

#### 3.5.3. Herbal Medicines—External Preparations

Seven studies used herbal medicine—external preparations to treat obesity (Table 8). Moszak et al. stated that administering 20 mL/day of *A. cruentus* (amaranth) seed oil or *B. napus* (rapeseed) oil generally improved insulin levels and percentage HDL compared to the control treatment [68]. However, all three groups showed significantly reduced weight, BMI, WC, HC, FM, lean body mass, visceral FM, and total body water percentage. Escalante et al. reported that topically applying Lipoxyderm, a lotion containing aminophylline, caffeine, Yohimbe, l-carnitine, and *C. asiatica* (gotu kola), twice daily for 28 days significantly decreased THC, thigh skinfold thickness, and thigh FM compared to the placebo [69]. Galvão Cândido et al. concluded that daily high-fat breakfasts containing 25 mL of extra virgin *O. europaea* (olive) oil over nine consecutive weeks led to higher fat loss [70]. Extra virgin olive oil also increased serum creatinine, decreased hepatic alkaline phosphatase, and generally reduced interleukin-1β (IL-1β) levels. Rezaei et al. found that consuming 20 g/day of *L. usitatissimum* (flaxseed) oil for 12 weeks resulted in greater weight loss and decreased WC than the placebo [71]. The intervention proved that flaxseed oil benefits patients with nonalcoholic fatty liver disease when combined with a low-energy diet and moderate physical activity. Lima et al. showed that after receiving 300 g of vegetables and legumes containing varying folate levels and a *Corylus* (hazelnut) capsule, women with overweight did not show weight loss but did show reduced beta-3 adrenergic receptor (*ADRB3*) gene methylation and malondialdehyde levels and increased in HDL-C and total antioxidant capacity [72].

Altogether, these studies show that external herbal medicine preparations can help reduce obesity. These studies mainly focused on oils and examined more than one factor. However, some results were not significant, and indicators showed less consistency across studies than in other fields.

## 4. Discussion

Obesity is a global burden transcending borders with continuously high prevalence rates [75]. While current technologies and synthetic medicines are being adopted to treat obesity, their related complications and safety issues are still being discussed. Traditional herbal medicines have arisen as effective agents to alleviate this multifactorial disease, and various studies have scrutinized the antiobesity effects of natural products. While many systemic reviews have examined the effects of natural products against obesity, none have systematically categorized natural drugs and mechanisms. In addition, this review is the most recent to assess extensive natural products. This review summarizes the effects and related mechanisms of each natural product studied in clinical trials. The natural products were classified into seven groups: natural compounds, foods, teas, fruits, extracts, decoctions, and external preparations. The mechanisms of the natural products were organized into lipid metabolism, anti-inflammation, antioxidant, appetite loss, and thermogenesis.

### 4.1. Antiobesity Mechanism

Based on the reviewed studies, natural products that demonstrated efficacy in alleviating obesity shared common mechanisms. Major mechanisms included lipid metabolism, anti-inflammation, antioxidation, appetite loss, and thermogenesis. The efficacy was evident in regulating lipid parameters, cytokines, hormones, or genes. By comprehensively understanding the efficacy and related mechanisms, this review extensively identified the potential effects of various natural products for treating obesity.

#### 4.1.1. Lipid Metabolism

**Figure 1:**
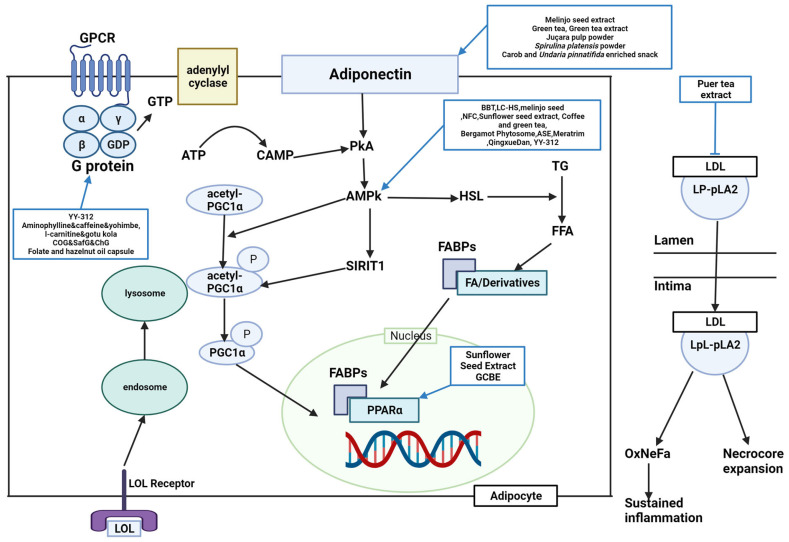
**Schematic diagram of lipid metabolism and the effects of natural products.** BBT, black soybean testa extract; LPL, lipoproteinlipase; HSL, hormone sensitive lipase; AMPK, adenosine monophosphate-activated protein kinase; NFC, *Nigella sativa* and *Trigonella foenum* graecum supplemented chapatis; LC-HS, *Lippia citriodora* L. and *Hibiscus sabdariffa* L; ASE, *Aster spathulifolius* Maxim extract; OPE, onion peel extract; CPT-1α, carnitine palmitoyltransferase I alpha; GCBE, green coffee bean extract; YY-312, *Imperata cylindrica* Beauvois, *Citrus unshiu* Markovich, and *Evodia officinalis* Dode; PPARα, peroxisome proliferator-activated receptor alpha; Lp-PLA2, lipoprotein-associated phospholipase A2; LDL, low-density lipoprotein; CoG, coconut oil group; SafG, safflower oil group; ChG, chia oil Group; GTP, guanosine triphosphate; ADRB3, A/T-, and β-adrenergic receptor 3; GPCR, G protein-coupled receptor; GTP, guanosine triphosphate; ATP, adenosine triphosphate; cAMP, cyclic adenosine monophosphate; AMPK, adenosine monophosphate-activated protein kinase; TG, triglycerides; FFA, free fatty acids; FABPs, fatty acid-binding protein; FA, fatty acid; OxNeFa, oxidized nonesterified fatty acids; PGC1α, peroxisome proliferator-activated receptor-gamma coactivator-1 alpha; PKA, protein kinase A; LpL-PLA2, lysophospholipid-associated phospholipase A2.

**Figure 2:**
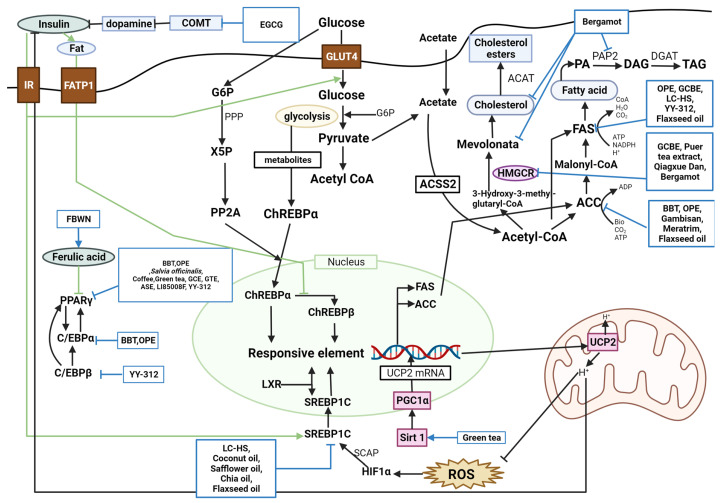
**Schematic diagram of glucose metabolism and the effects of natural products.** PPARγ, peroxisome proliferator-activated receptor gamma; BBT, black soybean testa extract; OPE, onion peel extract; GCBE, green coffee bean extract; GTE, green tea extract; ASE, *Aster spathulifolius* Maxim extract; LI85008F, *Moringa oleifera* leaf aqueous ethanol extract, *Murraya koenigii* (L.) Spreng. leaf aqueous ethanol extract, and *Curcuma longa* L. extract; YY-312, *Imperata cylindrica* Beauvois, *Citrus unshiu* Markovich, and *Evodia officinalis* Dode; ACC, acetyl-CoA carboxylase; ATP, adenosine triphosphate; ADP, adenosine diphosphate; C/EBPα, CCAAT-enhancer-binding protein alpha; FAS, fatty acid synthase; PA, phosphatidic acid; PAP2, type-2 phosphatidic acid phosphatase; DAG, diacylglycerol; DGAT, diacylglycerol-acyltransferase; TAG, triacylglycerol; LC-HS, *Lippia citriodora* L. and *Hibiscus sabdariffa* L.; NADPH, nicotinamide adenine dinucleotide phosphate; CoA, coenzyme A; C/EBPβ, CCAAT/enhancer-binding protein beta; HMGCR, HMG-CoA reductase; ACAT, acylCoA cholesterol acyl transferase; srebp-1c, sterol regulatory element-binding protein 1c; LXR, liver X receptor; FBWN, *Lactobacillus plantarum* fermented barley-wheat flour compound noodle; COMT, catechol-O-methyltransferase; IR, insulin receptor; FATP1, fatty acid transport protein 1; GLUT4, glucose transporter type 4; G6P, glucose-6-phosphate; Acetyl CoA, acetyl coenzyme A; ACSS2, acetyl-CoA synthetase 2; ChREBPα, carbohydrate response element binding protein α; ChREBPβ, carbohydrate response element binding protein β; UCP2, uncoupling protein 2; ROS, reactive oxygen species; HIF1α, hypoxia inducible factor 1; SCAP, stem cells from apical papilla; SIRT1, sirtuin-1; PGC-1α, peroxisome proliferator-activated receptor gamma coactivator 1-alpha; UCP2 mRNA, uncoupling protein 2 messenger RNA; PPP, phosphatidate phosphohydrolase; X5P, xylulose 5-phosphate; PP2A, protein phosphatase 2A.

Various studies identified lipid metabolism when discussing how the target compound works to treat obesity (Figure 1 and Figure 2). Forty-five studies were regarded to have a lipid metabolism pathway, although nine studies lacked an appropriate mechanism.

Lipid metabolism is classified into lipogenesis, lipolysis, and adipocyte differentiation, and the corroborated antiobesity effects are explained by suppressing lipogenesis, accumulation, and adipocyte differentiation and inducing lipolysis and fatty acid oxidation. Peroxisome proliferator-activated receptor gamma (PPARγ), acetyl-CoA carboxylase (ACC), CCAAT-enhancer-binding protein alpha (C/EBPα), CCAAT/enhancer-binding protein beta (C/EBPβ), fatty acid synthase (FAS), and sterol regulatory element-binding protein 1c (SREBP-1C) are lipogenic factors. Lipoprotein lipase (LPL), hormone-sensitive lipase (HSL), peroxisome proliferator-activated receptor alpha (PPARα), and adenosine monophosphate (AMP)-activated protein kinase (AMPK) are lipolysis factors. Several studies found these factors to regulate obesity complexly.

PPARγ was downregulated by BBT, OPE, common sage, coffee, green tea, GCBE, GTE, ASE, LI85008F, and YY-312 [11,22,25,35,39,41,57,61,64]. BBT, OPE, Gambisan, Meratrim, and flaxseed oil are associated with ACC inhibition [11,22,63,67,71]. BBT and OPE have been reported to downregulate C/EBPα [11,22]. FAS was inhibited by OPE, GCBE, LC-HS, YY-312, and flaxseed oil [22,40,61,62,71]. YY-312 downregulated C/EBPβ, thereby inhibiting adipocyte differentiation [61]. BBT upregulated lipolysis proteins such as LPL and HSL [11]. AMPK is predominantly associated with antiobesity metabolism. BBT, LC-HS, melinjo seed, NFC, sunflower seed extract, coffee and green tea, bergamot phytosome, ASE, LC-HS, Meratrim, Qingxue Dan, and YY-312 were reported to activate AMPK [11,18,20,21,28,35,47,57,61,62,63,65]. OPE upregulated carnitine palmitoyltransferase I alpha (CPT-1α) [22]. Sunflower seed extract and GCBE upregulated *PPARα* expression [28,40]. APN was upregulated by a carob- and wakame-enriched snack, melinjo seed, green tea, GTE, juçara pulp powder, and *S. platensis* powder, decreasing lipogenesis and inducing β-oxidation [13,20,36,41,51,54]. Pancreatic lipase was inhibited by PTE, mangosteen extract, Gambisan, and Qingxue Dan [45,52,65,67]. HMG-CoA reductase (HMGCR), a cholesterol synthesis enzyme, was inhibited by GCBE, PTE, and Qingxue Dan [40,45,65]. PTE acts as a noncompetitive inhibitor of lipoprotein-associated phospholipase A2 (Lp-PLA2) [45]. LC-HS, coconut oil, safflower oil, chia oil, and flaxseed oil suppressed SREBP-1C [62,71,74]. A carob- and wakame-enriched snack and Qingxue Dan activated the LDL receptor, inhibiting lipid synthesis [13,65]. YY-312, Lipoxyderm, coconut oil, safflower oil, chia oil, and folate and hazelnut oil capsules altered steps in the process in which activation of guanosine triphosphate (GTP)-binding proteins successively activates adenylate cyclase, cyclic AMP (cAMP), and protein kinase A (PKA), and lipase [61,69,72,74]. GCBE and bergamot inhibited the activation of acyl-CoA cholesterol acyl transferase (ACAT) [40,49]. GCBE upregulated carnitine palmitoyl transferase, a fatty acid oxidation enzyme [40]. FBWN increased ferulic acid, inhibiting lipid accumulation and regulating lipid metabolism [17]. High-dose GTE increased fat oxidation by inhibiting catechol-O-methyltransferase (COMT) [42]. Quercetin-rich onion powder altered the expression of genes related to fat metabolism, such as ADRB3, HSL, PPARγ, and uncoupling protein (UCP)-2 [24]. Standardized extracts of Malabar tamarind konjac regulated lipolysis by activating catecholamine signaling [26]. Green tea increased SIRT1-mediated PGC-1α activity, decreasing adipocyte differentiation, proliferation, and the expression of genes involved in lipogenesis [36]. Bergamot inhibited HMGCR and reduced cholesterol levels, mevalonate levels, and hepatic TG accumulation by inhibiting phosphatidate phosphohydrolase activity [49].

#### 4.1.2. Anti-Inflammation

**Figure 3:**
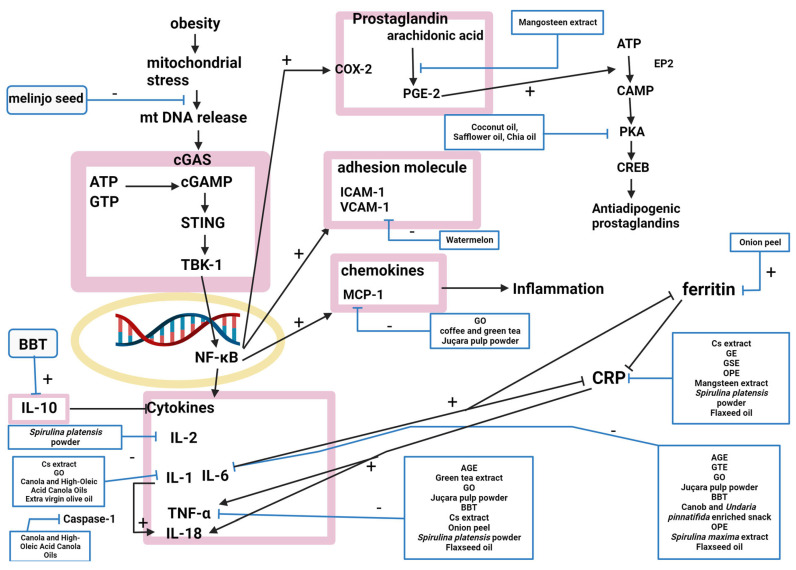
The nuclear factor-kappa B (NF-κB) signaling pathway was inhibited by AGE, GTE, GO, and juçara pulp powder, attenuating the production of proinflammatory cytokines and suppressing obesity-induced inflammation [9,41,50,51]. NF-κB inhibition decreases the circulating levels of proinflammatory cytokines, including tumor necrosis factor-α (TNF-α) and interleukin (IL)-6. IL-6 was decreased by AGE, BBT, a carob- and wakame-enriched snack, OPE, GTE, GO, juçara pulp powder, *S. maxima* extract, and flaxseed oil [9,11,13,22,41,50,51,53,71]. TNF-α was inhibited by AGE, BBT, artichoke extract, GSE, onion peel, GTE, GO, juçara pulp powder, *S. platensis* powder, and flaxseed oil [9,11,14,16,22,41,50,51,54,71]. Coffee and green tea, GO, and juçara pulp powder downregulated the expression of monocyte chemoattractant protein-1 (MCP-1), inhibiting monocyte adhesion [35,50,51]. Artichoke extract, GE, GSE, OPE, mangosteen extract, *S. platensis* powder, and flaxseed oil decreased high-sensitivity C-reactive protein (hsCRP) levels [14,15,16,22,52,54,71]. GO, canola oil, high-oleic-acid canola oil, and extra virgin olive oil reduced IL-1β [50,70,73]. BBT increased IL-10 [11]. Artichoke extract regulated IL-1 and interferon (IFN) [14]. Melinjo seed activated the cGMP-AMP (cGAMP) synthase-cGAMP-stimulator of interferon genes pathway by activating disulfide bond A oxidoreductase-like protein (DSBA-L) [20]. Onion peel decreased ferritin [22]. Watermelon decreased VCAM-1, intercellular adhesion molecule 1 (ICAM-1), and P-selectin, which attracts immune cells to damaged areas of the endothelium [29]. Mangosteen extract inhibited the conversion of arachidonic acid to prostaglandin E2 (PGE2) by altering cyclooxygenase (COX) and COX2 gene expression [52]. IL-2 was decreased by *S. platensis* powder [54]. Canola oil and high-oleic-acid canola oils inhibited inflammasome-mediated caspase-1 (CASP1) activity [73]. Coconut, safflower, and chia oils upregulated cAMP-dependent signaling pathways, which produce antiadipogenic prostaglandins that function in the adaptive reactions of cyclooxygenases [74].

Twenty-one natural products modulated the inflammation pathway, and twenty studies explained their mechanisms (Figure 3).

#### 4.1.3. Antioxidant

**Figure 4:**
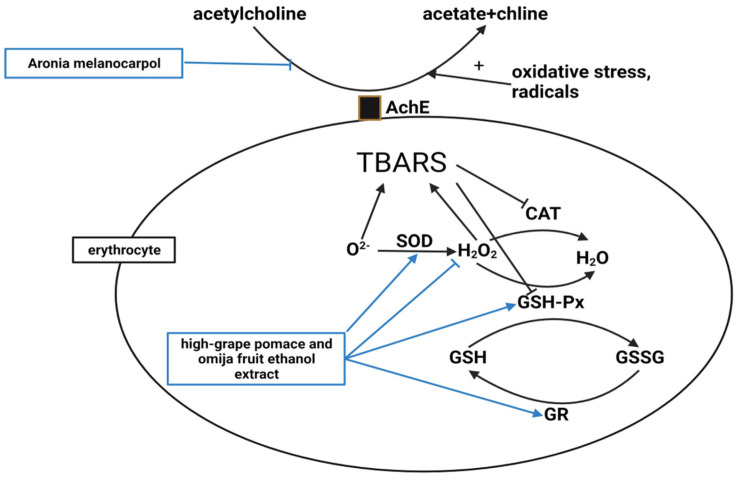
High oxidative stress and free radicals increase AChE activity. However, *A. melanocarpa* decreased the AChE activity in the erythrocyte membranes [46]. Superoxide dismutase (SOD) catalyzes the dismutation of the superoxide anion to H_2_O_2_, then catalase (CAT) and glutathione peroxidase (GSH-Px) degrade H_2_O_2_. GSH-Px also oxidizes reduced glutathione (GSH) to oxidized glutathione (GSSG), and GSSG is reduced to GSH by glutathione reductase (GR). A high-GO supplement elevated erythrocyte SOD, GSH-Px, and GR activities and lowered H_2_O_2_ levels [50]. Thiobarbituric acid reactive substances (TBARS), a marker of lipid peroxidation caused by oxidative injury, were also reduced by the high-GO supplement [50].

The next mechanism associated with antiobesity is antioxidation (Figure 4). Ten studies noted antioxidant effects, but only two discussed the antioxidant mechanism associated with their compound’s significant efficacy.

#### 4.1.4. Appetite Loss

**Figure 5:**
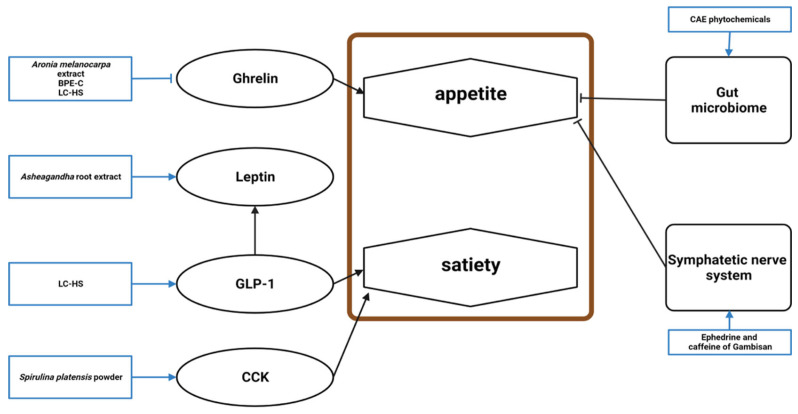
**Schematic diagram of the appetite mechanism in obesity and the effects of natural products.** BPE-C, bergamot polyphenol extract complex; LC-HS, *Lippia citriodora* L. and *Hibiscus sabdariffa* L.; GLP-1, glucagon-like peptide-1; CCK, cholecystokinin; CAE, caraway aqueous extract.

Twelve studies established a connection between appetite and the effects of natural products, and nine mentioned the mechanism underlying this effect (Figure 5). Among the three excluded studies, one did not show a significant appetite suppression effect but reported a related mechanism. Appetite loss manifests as increased anorexigenic factors and decreased orexigenic factors.

Ghrelin was lowered by *A. melanocarpa* extract, BPE-C, and LC-HS [46,48,62]. LC-HS increased glucagon-like peptide-1 (GLP-1), an anorexigenic incretin produced by the intestinal L-cells that stimulates insulin secretion and induces satiety [18,62]. Ashwagandha root extract reduced stress, restoring leptin levels, which suppresses food intake [56]. CAE phytochemicals, including limonene, γ-terpinene, trans-carveol, carvone, thymol, and carvacrol, improved the gastrointestinal microbiome to alter appetite [12]. The phenylalanine content of *S. platensis* powder may be responsible for cholecystokinin release, which affects the brain’s appetite center [54]. The ephedrine and caffeine in Gambisan reversed obesity by reducing food intake via the sympathetic nervous system [67].

#### 4.1.5. Thermogenesis

**Figure 6:**
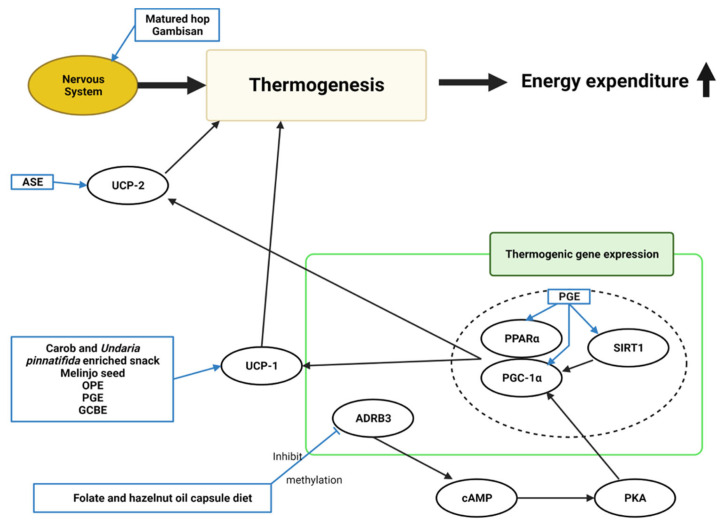
**Schematic diagram of the thermogenesis mechanism in obesity and the effects of natural products.** ASE, *Aster spathulifolius* Maxim extract; OPE, onion peel extract; PGE, *Platycodon grandiflorus* ethanol extract; GCBE, green coffee bean extract; UCP-1, uncoupling protein-1; UCP-2, uncoupling protein-2; ADRB3, adrenoceptor beta-3; cAMP, cyclic adenosine monophosphate; PKA, protein kinase A; PGC-1α, peroxisome proliferator-activated receptor γ coactivator 1α; PPARα, peroxisome proliferator-activated receptor alpha; SIRT1, sirtuin 1; ↑, increase.

Ten studies mentioned the relationship between thermogenesis and the effects of natural products, though one only stated the effects without explaining the mechanism (Figure 6). Increased thermogenic gene expression and factors caused the browning of white adipose tissues.

A carob- and wakame-enriched snack, melinjo seed, OPE, PGE, and GCBE induced uncoupling protein-1 (*UCP1*) in brown adipose [13,20,22,23,40]. ASE increased uncoupling protein-2 (*UCP2*) expression, increasing energy expenditure and consumption [57]. The sympathetic nervous system was considered related to energy expenditure through thermogenesis. Matured hop and Gambisan were believed to activate the nerve system [19,67]. PGE increased the expression of thermogenic-related genes, such as *SIRT1*, *PPARα*, and *PGC-1α* [23]. Folate and hazelnut oil capsules lowered *ADRB3* gene methylation levels [72]. The ADRB3 protein facilitates the catecholamine-induced activation of adenylate cyclase through the actions of G proteins. These mechanisms are involved in energy homeostasis by mediating thermogenesis.

### 4.2. Limitations

However, these studies had limitations. Some lacked information or were unconvincing. The study about DEA was conducted for a short period with few participants, so its results have low reliability [8]. Moreover, its study design was neither blind nor placebo-controlled. Further studies on a larger scale and with a longer observation period are needed to support their conclusions. In addition, several studies had many nonsignificant results, making it difficult to prove the efficacy of their natural products. Laboratory tests were left blank in the table when the obesity indicators were nonsignificant, the parameters were unrelated to obesity or lipid metabolism, or there were no serological indicators. Moreover, a few studies were the first clinical trials on their target compound [9,23,53,57,58,68,70,72]. Therefore, the drug dosage was determined based on the results of animal experiments since there were no human reference data, leading to relatively low confidence in their experimental results. Finally, some studies focused on obesity-related bioavailability, such as metabolic disorders, inflammatory status, or antioxidant capacity, rather than on obesity itself. The effects of IP-A and IP-B were mainly studied on postprandial blood glucose and peak insulin, and the outcome for obesity was just peripheral [66].

This review also had some limitations that should be addressed in future studies. First, it only selected clinical trials, excluding in vitro and in vivo studies. In addition, it only included studies written in English. Lastly, while it contained natural products of various origins, reviews on each referenced study were insufficient.

### 4.3. Well-Designed Studies in Antiobesity

Despite these shortcomings, this review included specific mechanisms of obesity and its treatments, specific features of obesity, and laboratory test results that decreased obesity. Among the clinical trials analyzed in this review, three with outstanding results are especially noted below. First, the effects of GCBE combined with an energy-restricted diet on lipid composition were examined in 64 women with obesity aged 20–45 years [40]. After eight weeks of taking 400 mg of GCBE, the intervention group showed significantly decreased BW, BMI, FMI, and WHR compared to the placebo group. These outcomes were supported by decreased serum TC, LDL, leptin, and FFA. In addition, this study investigated the change in serum APN levels with GCBE for the first time, showing they significantly increased in the experimental group.

Second, a mixture of grape pomace and omija fruit ethanol extracts was prepared to evaluate its effects on lipid profiles, inflammatory status, and antioxidant capacity [51]. The dose-dependent antiobesity effect was outstanding. This combination was examined in three different groups: high-dose GO (grape pomace extract (685 mg/day) + omija fruit extract (115 mg/day); *n* = 26), low-dose GO (grape pomace extract (342.5 mg/day) + omija fruit extract (57.5 mg/day); *n* = 26), and control (starch (4 g/day); *n* = 24). GO was provided in capsules, and participants were encouraged to take two capsules twice daily for 10 weeks. The high-dose GO supplement reduced TC, non-HDL-C, LDL-C, plasma ApoB, and plasma Lp(a) and increased ApoA-1. This study demonstrated that GO could be an advantageous natural product for improving dyslipidemia and metabolic disorders in individuals with overweight or obesity without side effects. It also served as a meaningful preliminary study to determine the GO dose.

Lastly, Meratrim is a mixture of extracts from *S. indicus* flower heads and mangosteen fruit rinds [66]. This study was a follow-up to evaluate the efficacy and tolerability of Meratrim, which was already proven effective against obesity in the previous study. Fifty-seven subjects were analyzed after taking a 400 mg Meratrim supplement twice daily for 16 weeks while consuming approximately 2000 kcal and walking 30 min daily for five days per week. Meratrim caused remarkable decreases in TG, LDL, and TC and increased glycerol production, AMPK, ACC phosphorylation, and HDL. The changes in these serological indicators led to reduced BW, BMI, and waist and hip size compared to the placebo group. Altogether, these findings indicate that this herbal formulation is effective and well tolerated in weight management in healthy individuals with overweight. Moreover, there were no adverse side effects.

It is evident that various studies have examined the effects of natural products on obesity. This review detailed the potential for the widespread use of natural products in treating obesity, which has not been reported in previous reviews on the same topic. Based on this review, further studies on safety, tolerability, and pharmacokinetics can be performed on these natural products to confirm their potential effectiveness.

## 5. Conclusions

This review comprehensively considered the effects of natural products against obesity by classifying sixty-two studies into various antiobesity mechanisms. Natural compounds, foods, tea, fruit, extracts, decoctions, and external preparations were found to show efficacy in lipid metabolism, anti-inflammation, antioxidation, appetite loss, and thermogenesis. Most studies showed positive effects in relieving the symptoms of obesity and demonstrated that natural products could be used as effective treatments for obesity. Therefore, herbal medicines are expected to be fully utilized in clinical obesity treatment. However, limitations remain in that some studies did not investigate efficacy or safety, and their nonsignificant results could be changed with precise control of drug dosages. Therefore, meta-analyses are needed to further examine their findings. Further studies are expected to refine the pharmacological effects of natural products for clinical use.

## Figures and Tables

**Table 1:** Single compound.

Compound	Study Design	Population	Status	Number	Outcome	Lab Test	Reference
Diethyl azelate	21 days prospective, before–after	17	Completed		Decreased obesity	↓ TC/HDL ratio, LDL/HDL ratio, noncholesterol HDL/HDL ratio	[8]

TC, total cholesterol; HDL, high-density lipoprotein; LDL, low-density lipoprotein. ↓, decrease.

**Table 2:** Foods.

Extract	Study Design	Population	Status	Number	Outcome	Lab Test	Reference
*Allium sativum* (aged garlic extract)	Double-blind, randomized, placebo-controlled clinical trial	48	Completed	NCT01959646	Decreased obesity	↓ LDL	[9]
*Citrus bergamia* (bergamot) and *Cynara cardunculus*	Double-blind placebo-controlled clinical trial	86	Completed	ISRCTN12833814	Decreased BW	↓ LDL-C, HDL-C, non-HDL-C, TC	[10]
*Glycine max* (L.) Merr (black soybean testa extract)	8-week planned, randomized, double-blind, placebo-controlled clinical trial	63	Completed	NCT02108691	Decreased obesity	↓ TG, LDL, non-HDL	[11]
*Carum carvi* L. (caraway aqueous extract)	Triple-blind, placebo-controlled clinical trial	60	Completed	NCT01833377	Decreased obesity, appetite		[12]
*Ceratonia siliqua* (carob) and *Undaria pinnatifida* (wakame) enriched snack	8-week, randomized, placebo-controlled clinical trial	32	Completed	NCT03420989	Decreased obesity	↓ TC, resistin levels, LDL-C	[13]
*Cynara scolymus* (artichoke) extract	Double-blind, placebo-controlled, randomized clinical trial	54			Decreased obesity, decreased BW and BMI	↑ HDL; ↓TC, TC/HDL, LDL, LDL/HDL, ApoB, ApoB/ApoA	[14]
*Allium sativum* (garlic extract)	Randomized double-blind placebo-controlled nutritional intervention clinical trial with two parallel arms	92		DRKS00010533	Decreased obesity	↓ LDL-C	[15]
*Vitis vinifera* L. (grape) seed extract	Randomized, double-blind, placebo-controlled clinical trial	40	Completed	IRCT2015073015968N3	Decreased obesity	↓ NPY	[16]
*Lactobacillus plantarum* fermented *Hordeum vulgare-Triticum aestivum* (barley-wheat) flour compound noodle	Single-blinded, controlled, parallel clinical trial	30	Completed	ChiCTR1800019614	Decreased obesity	↓ TG	[17]
*Lippia citriodora* (lemon beebrush) and *Hibiscus sabdariffa* (roselle)	8-week, randomized, double-blind, placebo-controlled clinical trial	54	Completed		Decreased obesity, appetite	↓ Leptin, resistin	[18]
Matured *Humulus lupulus* L. (hops)	Randomized, double-blind, placebo-controlled parallel-arm clinical trial	178	Completed	UMIN000014185	Decreased BF		[19]
*Gnetum gnemon* Linn (melinjo) seed	Prospective, randomized, parallel, double-blind, placebo-controlled clinical trial	42	Completed	UMIN000025643	Increased APN multimerization	↑ HMW/total APN ratio	[20]
*Nigella sativa* (black seed or jintan hitam) and *Trigonella foenum-graecum* (fenugreek) supplemented chapatis	12-week prospective, before–after clinical trial	40	Completed		Decreased obesity	↓ TC, non-↑ HDL-C, VLDL, TG, ↓ HbA1C, FPG	[21]
*Allium cepa* L. (onion) peel	Randomized, double-blind, placebo-controlled clinical trial	61			Decreased obesity	↑ PUFA n-6 ↓ PUFA n-3	[22]
*Platycodon grandiflorus* (balloon flower) ethanol extract	Single-center, randomized, double-blind, placebo-controlled clinical trial	72	Completed		Decreased obesity	PGE571: ↓ leptin. PGE2855: ↓ L:A ratio	[23]
Quercetin-rich *Allium cepa* L. (onion) powder	Randomized, double-blind, placebo-controlled, parallel-group clinical trial	54	Completed	UMIN000033410	Subjects with lower HDL-C: decreased VFA.		[24]
*Salvia officinalis* (common sage)	Randomized triple-blinded placebo-controlled clinical trial	60	Completed	IRCT201504146917N2	Decreased obesity		[25]
*Garcinia cambogia* (Malabar tamarind) and *Amorphophallus konjac* (konjac)	Prospective, nonrandomized controlled intervention clinical trial	214	Completed		Decreased weight	↓ Cholesterol, TG	[26]
*Stevia rebaudiana* (stevia)	Randomized, three-arm, single-blinded crossover clinical trial	30	Completed	NCT01115088	Decreased energy intake		[27]
*Helianthus annuus* (sunflower) seed extract	Randomized, placebo-controlled, double-blind, parallel-group clinical pilot study	46	Completed		Decreased obesity	↓ Cholesterol, long-lasting LDL	[28]
*Citrullus lanatus* (watermelon)	Randomized 2-arm design with a single 6-week intervention period	45	Completed	NCT04015544	Decreased obesity		[29]
*Caulerpa racemosa* (green algae)	Randomized, double-blind, placebo-controlled clinical trial	74	Completed	NCT05037591	Decreased obesity	↑ HDL, proliferator-activated receptor-γ coactivator α (PGC-1α); ↓ TC, TG	[30]
*Cyperus rotundus* rhizome extract	Randomized, double-blind, parallel-group, placebo-controlled pilot study	30	Completed	CTRI/2014/05/004633	Decreased waist circumference and BMI	↓ TC, TG, LDL, VLDL; ↑ HDL	[31]
*Garcinia cambogia* (Malabar tamarind) extract	Open-label clinical study	100	Completed		Improved anthropometric and metabolic state	↓ LDL; ↑ HDL	[32]
*Hydrangea serrata* (Thunb.) Ser. leaf extract	Randomized, double-blind, placebo-controlled clinical trial	93	Completed	KCT0005594	Decreased overweight	↓ LDL, TG	[33]
*Citrus reticulata* (immature poken) extract	Randomized, placebo-controlled clinical trial	20	Completed	CMUH103-REC2-040	Decreased weight and fat metabolism by suppressing adipogenesis	↓ LDL, TG, TC	[34]

LDL, low-density lipoprotein; BW, body weight; HDL, high-density lipoprotein; TC, total cholesterol; TG, triacylglycerols (triglyceride); ApoB, apolipoprotein B; ApoA, apolipoprotein A; NPY, neuropeptide Y; BF, body fat; HMW, high molecular weight; APN, adiponectin; VLDL, very-low-density lipoprotein; PUFA, polyunsaturated fatty acid; L:A, leptin:adiponectin; VFA, visceral fat area; FPG, fasting plasma glucose; ↓, decrease; ↑, increase.

**Table 3:** Teas.

Tea	Study Design	Population	Status	Number	Outcome	Lab Test	Reference
*Coffea arabica* (coffee), *Camellia sinensis* (green tea)	Cross-sectional, brief-type self-administered diet history questionnaire	232	Completed		Decreased BW and BMI		[35]
Coffee, green tea	Cross-sectional, Japan multi-institutional collaborative cohort study	3539	Completed		Coffee: decreased VAT, metabolic syndrome		[38]
Decaffeinated green coffee bean extract	Randomized, double-blind, placebo-controlled trial	43	Completed	NCT02764957	Decreased obesity and appetite		[39]
Green coffee bean extract	Randomized, double-blind, placebo-controlled clinical trial	64	Completed		Decreased obesity	↑ Serum adiponectin; ↓ total serum cholesterol, LDL, FFA, leptin	[40]
Green tea	10-week randomized, placebo-controlled trial	30	Completed	NCT04950062	Increased metabolic status	↑ PGC-1α	[36]
Green tea	Randomized, double-blind, placebo-controlled clinical trial	124	Completed		Decreased BF		[37]
Green tea extract	Double-blinded placebo-controlled trial	45	Completed	IRCT20151025024699N3	Decreased obesity	↑ Adiponectin, irisin	[41]
High-dose green tea extract (epigallocatechin gallate)	Randomized, single-center, placebo-controlled, double-blind study	77	Unknown	NCT02147041	Decreased weight	↑ Adiponectin; ↓ cholesterol, LDL, ghrelin	[42]
Kosen-cha	12-week, prospective, before–after study	6	Completed		Decreased obesity	↓TG, ↑insulin sensitivity	[43]
Oolong tea	14-day, placebo-controlled, double-blind, crossover intervention trial	12	Completed		Increased FO		[44]
Puer tea extract	Randomized, double-blind, placebo-controlled clinical trial	59	Completed	NCT03613688	Decreased obesity	↓ Cholesterol	[45]

BW, body weight; BMI, body mass index; VAT, visceral adipose tissue; LDL, low-density lipoprotein; FFA, free fatty acid; PGC-1α, proliferator-activated receptor gamma coactivator 1-alpha; BF, body fat; FO, fat oxidation; ↓, decrease; ↑, increase.

**Table 4:** Fruits.

Extract	Study Design	Population	Status	Number	Outcome	Lab Test	Reference
*Aronia melanocarpa* extract	Placebo-controlled trial	77	Completed		Decreased cholinesterase activity	↑ HDL, cholesterol, TAC “fast” parameter; ↓ TC, LDL, TG, TAC “slow” parameter, lipid peroxidation, cholesterol in the erythrocyte membranes	[46]
*Citrus bergamia* (bergamot) phytosome	Randomized, double-blind, placebo-controlled trial	64	Completed		Decreased VAT	↓ TC, LDL, ApoB, LDL/HDL; ↑ ApoA/HDL	[47]
*Citrus bergamia* (bergamot) polyphenol extract-complex	Randomized, double-blind, placebo-controlled trial	45	Completed	UNICZ Trial No. 182/2016	Decreased weight	↓ TC, LDL, TAG, serum leptin, serum ghrelin; ↑ HDL, serum adiponectin	[48]
*Citrus bergamia* (bergamot)	Randomized, double-blind, placebo-controlled trial	98	Completed		Decreased cholesterol and BW	↓ LDL	[49]
Grape pomace and *Schisandra chinensis* (omija) fruit ethanol extract	Randomized, double-blind, placebo-controlled trial	76	Completed		Decreased obesity-related dyslipidemia	High GO: ↑ ApoA-1; ↓ TC, non-HDL-C, LDL-C, plasma ApoB, Apo B/ApoA-1 ratio, plasma Lp(a)	[50]
*Euterpe edulis* (juçara) pulp powder	Randomized, double-blind trial	35	Completed	RBR-5RXR2B	Decreased obesity	↑ HDL-C, serum adiponectin; ↓ TC, LDL, TAG, L:A ratio	[51]
*Garcinia mangostana* (mangosteen) extract	26-week prospective randomized, controlled, parallel-group study	20	Completed	NCT02823561	Decreased weight	↓ HDL	[52]

HDL, high-density lipoprotein; TAC, total antioxidant capacity; TC, total cholesterol; LDL, low-density lipoprotein; TG, triacylglycerols; VAT, visceral adipose tissue; ApoB, apolipoprotein B; ApoA, apolipoprotein A; TAG, triacylglycerols; BW, body weight; ApoA-1, apolipoprotein A-1; Lp(a), lipoprotein(a); L:A ratio, leptin-to-adiponectin ratio; ↓, decrease; ↑, increase.

**Table 5:** Herbal medicines—single extracts.

Extract	Study Design	Population	Status	Number	Outcome	Lab Test	Reference
*Withania somnifera* (ashwagandha) root extract	Double-blind, randomized, placebo-controlled trial	50	Completed		Decreased BW	↓ Mean FCQ scores, mean TFEQ score	[56]
*Aster spathulifolius* Maxim	Randomized, double-blind, placebo-controlled clinical trial	41	Completed		Decreased BW and FM	↑ LDL	[57]
Lipigo^®^	Randomized, double-blinded, placebo-controlled clinical trial	WLP: 98 P-WLP: 73	Completed	NCT03554525	Decreased BW, rebound effect		[58]
*Rhus coriaria* L. powder ethanolic extract	Randomized, double-blind, placebo-controlled clinical trial with two arms	70	Completed	NCT02295293	Increased ApoA-1 and HDL	↑ HDL, serum Apo-A1	[59]
*Spirulina maxima* extract	Randomized Double-Blind Placebo-Controlled Trial	50	Completed	NCT02575690	Decreased obesity	↓ LDL	[53]
*Spirulina platensis* powder	Randomized, double-blinded, placebo-controlled clinical trial	38	Completed	NCT02993627	Decreased obesity	↓ TG	[54]
*Zataria multiflora* Boiss with or without oxymel	Randomized, controlled, triple-blind Trial	92	Completed	IRCT20171220037976N1	Decreased obesity		[55]

BW, body weight; FCQ, Food Cravings Questionnaire; TFEQ, Three-Factor Eating Questionnaire; FM, fat mass; LDL, low-density lipoprotein; ApoA-1, apolipoprotein A-1; HDL, high-density lipoprotein; TG, triglycerides; ↓, decrease; ↑, increase.

**Table 6:** Herbal medicines—decoctions.

Drug	Study Design	Population	Status	Number	Outcome	Lab Test	Reference
Euiiyin-tang	Randomized, double-blind, placebo-controlled, multicenter trial	149	Completed	NCT01724099	Decreased obesity		[60]
Gambisan	Double-blinded, randomized, placebo-controlled, phase 2 trial	205	Completed		Decreased obesity and appetite		[67]
*Imperata cylindrica* Beauvois, *Citrus unshiu* Markovich, and *Evodia officinalis* Dode (YY-312)	Randomized, double-blind, placebo-controlled, parallel-group clinical trial	60	Completed	KCT0001225	Decreased BFM		[61]
*Lippia citriodora* (lemon beebrush) and *Hibiscus sabdariffa* (roselle; LC-HS)	Double-blind, placebo-controlled, randomized trial	56	Completed	P201731147	Decreased obesity		[62]
Meratrim	Randomized, double-blind, placebo-controlled trial	57	Completed	CTRI/2014/07/004727	Decreased obesity and appetite	↑ Glycerol production, AMPK, ACC phosphorylation, HDL; ↓ TG, TC, LDL	[63]
*Moringa oleifera* leaf aqueous ethanol extract, *Murraya koenigii* (L.) Spreng. leaf aqueous ethanol extract, and *Curcuma longa* L. extract (LI85008F)	Randomized, double-blind, placebo-controlled trial	140	Completed	C007185	Decreased weight	↑ HDL; ↓ LDL, VLDL, TC, TG	[64]
Qingxue Dan	Randomized, double-blinded, placebo-controlled trial with parallel arms	26	Completed		Decreased obesity	↓ TG	[65]
White mulberry, white bean extract, and green coffee (IP-A and IP-B)	Randomized, double-blind, placebo-controlled, crossover trial	Study 1: 32 Study 2: 150	Completed	PCT/IB2015/052650	Decreased obesity		[66]

BFM, body fat mass; AMPK, AMP-activated kinase; ACC, acetyl CoA carboxylase; HDL, high-density lipoprotein; TC, total cholesterol; LDL, low-density lipoprotein; VLDL, very-low-density lipoprotein; TG, triglyceride; ↓, decrease; ↑, increase.

**Table 7:** Constituents of the decoctions.

Drug	
Euiiyin-tang	*Ephedra sinica* Stapf, *Angelica gigantis* Radi, *Atractylodis rhizoma* Alba, *Coicis semen, Cinnamomi cortex*, *Paeonia lactiflora*, and *Glycyrrhiza uralensis*.
Gambisan	The herbal part of *Ephedra intermedia* Schrenk, *Gypsum Fibrosum*, the rhizome part of *Atractylodes lancea* DC, and the leaf part of *Thea sinensis* L.
LC-HS	Combination of polyphenolic extracts from *Lippia citriodora* L. and *Hibiscus sabdariffa* L.
YY-312	Herbal extract powder from *Imperata cylindrica* Beauvois, *Citrus unshiu* Markovich, and *Evodia officinalis* Dode.
Meratrim	A blend of two plant extracts obtained from *Sphaeranthus indicus* flower heads and *Garcinia mangostana* fruit rinds.
LI85008F	Six parts *Moringa oleifera* leaf aqueous ethanol extract, three parts *Murraya koenigii* (L.) Spreng. leaf aqueous ethanol extract, and 1 part *Curcuma longa* L. extract.
Qingxue Dan	Herbal formula consisting of radix of *Scutellaria baicalensis* Georgi, rhizoma of *Coptis japonica* Makino, cortex of *Phellodendron amurense* Ruprecht, fructus of *Gardenia jasminoides* Ellis, and rhizoma of *Rheum palmatum* Linne.
IP-A and IP-B	IP-A: A mixture of *Morus alba* (white mulberry), *Phaseolus vulgaris* (white bean) extract, and *Coffea arabica* (green coffee). IP-B: A mixture of white mulberry, white bean extract, and green coffee supplemented with inulin and glucomannan.

**Table 8:** Herbal medicines—external preparations.

Drug	Study Design	Population	Status	Number	Outcome	Lab Test	Reference
*Amaranthus cruentus* (amaranth) seed oil and *Brassica napus* (rapeseed) oil	Randomized, double-blind, controlled trial with three parallel arms	81	Completed		Decreased obesity		[68]
Aminophylline, caffeine, Yohimbe, l-carnitine, and *Centella asiatica* (gotu kola; Lipoxyderm)	28-day, double-blind, placebo-controlled, within-group study	7	Completed		Decreased thigh circumference, skinfold thickness, and FM		[69]
Canola oil, oleic, and DHA; *Zea mays* (corn)/*Carthamus tinctorius* (safflower) oil; and *Linum usitatissimum* (flax)/safflower oil	Randomized, crossover, controlled feeding study	101	Completed	NCT01351012	Decreased abdominal FM	↑ Plasma oleic acid; ↓ android FM, android-to-gynoid FM ratio; canola oleic oil: ↓ TG	[73]
*Cocos nucifera* (coconut) oil, *Carthamus tinctorius* (safflower) oil, *Salvia hispanica* (chia) oil	Randomized, double-blind, placebo-controlled clinical trial	75	Completed	RBR-36bjsc	Decreased obesity	Chia oil: ↑ HDL-C; ↓ cholesterol, LDL-C, and TG	[74]
Extra virgin *Olea europaea* (olive) oil	9-week, randomized, double-blind, placebo-controlled clinical trial	54	Completed		Decreased obesity.		[70]
*Linum usitatissimum* (flax) seed oil	Randomized, double-blind, placebo-controlled clinical trial	68	Completed	IRCT 2016011125957 N1	Decreased weight		[71]
Folate and *Corylus* (hazelnut) oil capsules	Double-blind, placebo-controlled intervention study	40	Completed	NCT02846025	Decreased obesity	↑ HDL; ↓ LDL. Group 1: ↑ HDL. Group 3: ↓ WHtR, LDL, and total fat intake.	[72]

FM, fat mass; TG, triglycerides; HDL, high-density lipoprotein; LDL, low-density lipoprotein; WHtR, waist-to-height ratio; ↓, decrease; ↑, increase.

## Data Availability

Not applicable.
